# Assessing the cost implications of integrating and scaling up HIV services for key populations in Kenya and Malawi

**DOI:** 10.1093/heapol/czaf067

**Published:** 2025-10-31

**Authors:** Andrea Salas-Ortiz, Marjorie Opuni, José Luis Figueroa, Jorge Eduardo Sánchez-Morales, Louis Masankha Banda, Alice Olawo, Spy Munthali, Julius Korir, Meghan DiCarlo, Sergio Bautista-Arredondo

**Affiliations:** Centre for Health Economics, University of York, York YO10 5DD, United Kingdom; Independent Consultant, Geneva 1211, Switzerland; Center for Evaluation Research and Surveys (CIEE), National Institute of Public Health (INSP), Cuernavaca, Morelos 62100, México; Economic Analysis and Health Research Unit, Ministry of Health, Mexico City 11800, Mexico; FHI 360, Lilongwe 1000, Malawi; FHI 360, Nairobi 00623, Kenya; University of Malawi, Zomba 2060, Malawi; Kenyatta University, Nairobi 00100, Kenya; FHI 360, Washington, DC 20037, United States; Center for Evaluation Research and Surveys (CIEE), National Institute of Public Health (INSP), Cuernavaca, Morelos 62100, México; Economic Analysis and Health Research Unit, Ministry of Health, Mexico City 11800, Mexico

**Keywords:** HIV, key populations, total costs, service scale-up, integration, efficiency

## Abstract

Limited research has been conducted on strategies to improve the efficiency of HIV services for key populations (KPs). This study investigates ways to enhance healthcare delivery efficiency, focusing on HIV services for KPs. We explore two strategies: expanding service volume and offering multiple HIV services within a single health facility. Using data from the Linkages Across the Continuum of HIV Services for Key Populations Affected by HIV (LINKAGES) program in Kenya and Malawi, we exploit the variation in services provided to assess correlations between different service delivery configurations and their costs. We apply log-log fixed-effects regression models to analyze relationships between the total costs of four HIV services and the volume and range of services delivered. We find that service volume increases correlate with higher total costs, albeit less than proportionally, consistent with possible economies of scale. Negative correlations between service integration and total costs suggest that integrating HIV services for KPs could lead to reduced total costs for some service combinations. These results indicate potential strategies to increase the efficiency of HIV services for KPs, which can inform strategic planning and program execution in Kenya, Malawi, and similar countries.

Key messages

- In the context of limited and decreasing resources for HIV programs, evidence-based strategies to improve service delivery efficiency are paramount.
- Expanding service volume shows potential for efficiency gains through economies of scale.
- Strategic integration of HIV services may improve efficiency for certain service combinations.

## Introduction

The persistent disparities in access to essential human immunodeficiency virus (HIV) services for key populations (KPs) at higher risk of HIV infection, such as sex workers (SW), men who have sex with men (MSM), and transgender women (TGW), represent a significant global health challenge (UNAIDS 2023). These populations face unique barriers to accessing HIV services due to stigma, legal challenges, and social marginalization (Kavanagh *et al.* 2021). Additionally, underfunding of HIV services for these groups is also a significant factor worldwide (UNAIDS 2023, Eiling 2024). Global guidance outlines clear recommendations for a comprehensive approach to HIV services for KPs, encompassing clinical services (World Health Organization 2016) and structural interventions (Laga and Vuylsteke 2011, Beattie *et al.* 2012, Vassall *et al.* 2014), delivered by community-based and community-led organizations (World Health Organization 2016). The global goal of ending the AIDS epidemic by 2030 cannot be achieved without a substantial expansion in access to HIV services for KPs (DiCarlo *et al.* 2022). Yet, decreasing global HIV financial resources raises serious concerns about funding availability for these essential, yet consistently underfunded services (UNAIDS 2023).

This paper investigates possible strategies for enhancing health service delivery efficiency, focusing on HIV services for KPs. Two potential strategies are examined (Siapka *et al.* 2014). First, efficiency could potentially be enhanced by expanding the service volumes of facilities providing HIV services to KPs, diluting fixed costs over a higher volume of services, and decreasing average costs per service. Additionally, larger service volumes could increase staff specialization and productivity (Bernet and Singh 2015, Getzen and Kobernick 2022), contributing to increased efficiency (Schneider *et al.* 2008). Second, offering multiple HIV services to KPs within a single health facility could improve efficiency by leveraging service integration benefits. This strategy implies that expanding the set of interventions offered along with one intervention could result in a reduction of its total costs (McRae *et al.* 2020, Freeman *et al.* 2021).

Prior research suggests that increased service volume can enhance the efficiency of HIV services for KPs (Dandona *et al.* 2005, Guinness *et al.* 2005, Marseille *et al.* 2007, Chandrashekar *et al.* 2010, Lépine *et al.* 2015). While research specifically examining the efficiency of integrating KP HIV services is limited, systematic reviews examining the integration of HIV and non-HIV services for the general population have found that the costs of integrated services tend to be lower (Sweeney *et al.* 2012, Obure *et al.* 2016, Bulstra *et al.* 2021). To our knowledge, no previous studies have examined how simultaneously integrating KP HIV services and scaling up their volume affects total service costs. In this paper, we explore these dynamics.

We use data from the Linkages Across the Continuum of HIV Services for Key Populations Affected by HIV (LINKAGES) program in Kenya and Malawi. Local community-based and KP-led organizations, termed implementing partners (IPs), delivered HIV services to KPs at standalone facilities called drop-in centers (DICs). In Kenya and Malawi, DICs delivered clinical HIV services to KPs, including HIV testing services (HTS), antiretroviral treatment (ART), sexually transmitted infection (STI) screening, management of sexual violence (MSV), sexual and reproductive health (SRH) services, pre-exposure prophylaxis (PrEP), and post-exposure prophylaxis (PEP). While HTS, ART, STI screening, and MSV were provided in almost all DICs, SRH, PrEP, and PEP service availability varied across DICs. This variation in service provision created a unique opportunity to assess correlations between different service delivery configurations and their costs. We apply log-log fixed-effects regression models to analyze the relationship between total costs of HTS, ART, STI screening, and MSV and the volume of services delivered. This analysis controls for service volume increases and other clinical HIV services delivered within a facility. We then predict average total implementation costs of HTS, ART, STI screening, and MSV under different scenarios to identify the most cost-efficient service delivery options. The scenarios we consider include: the integration of SRH, PrEP, or PEP services; and scenarios without integration of any of these services.

## Materials and methods

### The LINKAGES program

The LINKAGES program, operational from 2014 to 2021, was financed by the United States Agency for International Development (USAID) through the United States President's Emergency Plan for AIDS Relief (PEPFAR) and led by FHI 360. For a detailed overview of the LINKAGES program structure, see Supplementary Material S1. KP-led organizations delivered HIV services in DICs and in communities, while LINKAGES country offices provided on-the-ground program management, capacity-building, and technical support. US-based LINKAGES headquarters supplied funding, high-level program guidance, and technical assistance.

Alongside clinical HIV services, all DICs provided nonclinical services, including KP empowerment and engagement activities, structural services like stigma reduction, and peer outreach. The LINKAGES program also included pre-service delivery activities, like mapping and KP size estimation, as well as ongoing above-service management and monitoring.

### Data and sample

The data analyzed in this study, described elsewhere (Opuni *et al.* 2023a, 2023b), were compiled for US Government fiscal years (USG-FYs) 2018 and 2019 across all levels of the LINKAGES program in Kenya and Malawi. This included data from DICs, IPs, country offices, and headquarters. Data on program inputs, prices, expenditures, and outputs were collected retrospectively for FY 2018 and prospectively for FY 2019. We classified support from headquarters and country offices as “above-service delivery” inputs and startup activities as “pre-service delivery” inputs. The “service delivery” inputs encompassed IP and DIC personnel, peer workers, clinical supplies, utilities, transportation, equipment, and training (see Supplementary Material S2 for a comprehensive list of inputs). Our sample included 30 DICs in Kenya and 15 DICs in Malawi, with observations from fiscal years (FY) 2018 and 2019 (90 observations in total).

### Cost measurement

The methodology for estimating the costs of the clinical services delivered by DICs has been elaborated in previous publications (Opuni *et al.* 2023a, 2023b). We estimated economic costs from the provider’s perspective, capturing the value of all resources used to provide services, regardless of whether they resulted in expenditures for the LINKAGES program. We derived total annual costs per DIC through a multi-step process. Above-service delivery and pre-service delivery costs were distributed equally across DICs. IP costs for staff, other recurrent inputs, equipment, transportation, and training were apportioned to DICs based on the number of DICs per IP or staff time weights. To estimate total costs for each clinical HIV service—HTS, ART, STI screening, MSV, SRH, PrEP, and PEP—we added service-specific input costs to indirect costs, comprising above-service delivery, pre-service delivery, and overhead IP and DIC costs. We allocated indirect costs using weights derived by combining annual number of clients per clinical service over total DIC clients and the proportion of staff and peer worker time dedicated to each clinical service. This dual weighting approach was used to ensure that indirect costs reflect both service volume and human resource intensity. Condom and lubricant promotion costs were distributed proportionally across clinical services, as no output data were available. All costs are presented in 2019 U.S. dollars (US$).

### Econometric analysis

The dependent variables in our study were the annual total costs of HTS, ART, STI screening, and MSV for both FYs. We modeled the total cost of clinical service *i* at DIC *k* as a function of service volume, *qi*. Marginal costs are captured by the function *C*(.):


(1) $$C_{ik}=C(q_{ik})$$

We estimated cost functions for the four HIV clinical services delivered in almost every DIC (*i* = HTS, ART, STI screening, and MSV). The following specification was used, informed by past literature (Weaver and Deolalikar 2004):


(\hbox2) $$\begin{array}{rl} \ln\;(TC_{ikt}) & =\alpha_{i}+\beta_{1}\ln(q_{ikt})+\underset{\;j\neq i}{\sum }\;\gamma_{j}\ln(q_{\;jkt})\\ & \;+\gamma_{2}\mathrm{SRH},\mathrm{PrEP},\mathrm{PE}P_{kt}+\delta_{1}x_{kt}+\theta_{k}+\tau_{t}+\varepsilon_{ikt} \end{array}$$

In Equation (2), $\;TC_{ikt}$ represents the total cost of HIV clinical service *i* at DIC *k* in FY *t.* The volume of services provided for the same intervention *i* provided by DIC *k* in FY *t* is depicted by $q_{ikt}$. The coefficient $\beta_{1}$ represents the change in total cost of clinical service *i* when service volume *qi* increases at DIC *k*. Values of $\beta_{1}$ < 1 suggest potential economies of scale. Service integration effects are proxied by the volume of other distinct HIV clinical services offered at a given DIC (23–26). *j* represents the set of other interventions provided in the same DIC *k* and during the same FY *t,* where i≠j. For example, without loss of generality, when estimating the total cost function for HTS, which is clinical service *i*, $q_{j}$ represents the volume of the other interventions separately: ART, STI screening, and MSV services provided by the same DIC *k* in the same FY *t*. Negative $\gamma_{j}$ coefficients are indicative of potential economies of scope. The vector $\mathrm{SRH},\mathrm{PrEP},\mathrm{PE}P_{kt}$ indicates whether DIC *k* provides SRH, PrEP, and PEP services; any combination thereof, or none of these interventions.

Equation (2) controls for input-cost variation across DICs using a staff price index for each DIC, represented by *x*. In healthcare, a primary issue when estimating cost functions is the lack of factor price data (Zweifel *et al.* 2009). Since clinical and recurrent supply prices were consistent across DICs due to central purchasing, staff wages were the only input price with variation. We created a staff salary index, defined as the geometric mean of monthly salaries of all DIC staff that better accounts for salary variation between staff types. We also control for time-invariant unobservable factors potentially correlated with service volume and total costs within DICs (fixed effects), $\theta_{k}$, and potential aggregate shocks affecting the total costs of all DICs simultaneously, $\tau_{t}$. The term $\varepsilon_{ikt}$ represents the model’s idiosyncratic error. Due to the skewness of cost data, we expressed total costs and service volume in natural logarithms. In all models, we clustered standard errors at the DIC level and formally tested whether economies of scope coefficients differed from zero.

Finally, using the coefficients estimated from Equation (2), we predicted total costs for each service under different SRH, PrEP, and/or PEP service delivery scenarios. Since predictions with logged dependent variables require a special procedure to retransform the dependent variable into natural units (dollars), we applied Duan’s smearing method to remove the retransformation bias, following the literature (Weaver and Deolalikar 2004, Guinness *et al.* 2007, Cameron and Trivedi 2010). We conducted analyses using “Stata18” (StataCorp 2023).

## Results

### Cost description

Table 1 displays descriptive data on total annual costs, average costs, and numbers of HTS, ART, STI screenings, and MSV services (see Supplementary Material S3 for results by country). Annual average DIC total costs for HTS, ART, STI screening, and MSV were $72 397, $55 679, $59 804, and $10 827, respectively. Mean average costs per service were $68, $39, and $716 for HTS, STI screening, and MSV, respectively, and $847 per ART patient. In terms of DIC characteristics, about half of the DICs provided SRH, PrEP, and PEP services. Regarding KPs served, 42% of DICs catered to more than one KP, 47% served only FSWs, 4% served only MSM, and 7% exclusively MSW. Two-thirds of DICs were in Kenya.

**Table 1.** DIC total costs, average costs, and number of services delivered for HTS, ART, STI screening, and MSV.

	Total cost (2019-USD)				Average cost^a^ (2019-USD)				Number of services provided			
	HTS^b^	ART^c^	STI^d^	MSV^e^	HTS	ART	STI	MSV	HTS	ART	STI	MSV
*n*	90	90	90	90	90	89	90	87	90	89	90	90
Mean	72 397	55 679	59 804	10 827	68.4	847	39.2	716	1888	112	2273	80
Median	60 373	51 328	53 395	8536	44.5	531	25.2	205	1586	89	2012	30
Min	4452	5799	21 550	582	10	259	4.92	17.4	76	2	398	0
Max	263 374	150 863	140 580	35 501	332	5785	188	18 028	9253	346	6880	752
**DIC characteristics**					**Absolute frequency**				**Relative frequency (%)**			
Offer SRH services					51				57			
Offer PrEP services					51				57			
Offer PEP services					46				51			
Cover more than one KP					38				42			
Cover FSW only					42				47			
Cover MSM only					4				4			
Cover MSW only					6				7			
Kenya					60				67			
Malawi					30				33			

*n* = number of observations. USD = US dollars. Min = minimum value. Max = maximum value. ^a^The average cost was calculated by dividing the total cost by the number of services provided. ^b^Number of HIV tests delivered to KPs. ^c^Number of KPs who received antiretroviral treatment. ^d^Number of STI screening services delivered to KPs. ^e^Number of management of sexual violence services provided to KPs. FSW, Female sex workers. MSM, men who have sex with men. MSW, Male sex workers. HTS, HIV testing services. ART, antiretroviral treatment. STI, sexually transmitted infections. MSV, management of sexual violence. SRH, sexual and reproductive health. PrEP, pre-exposure prophylaxis. PEP, post-exposure prophylaxis. DIC, drop-in-center.

**Figure 1.**
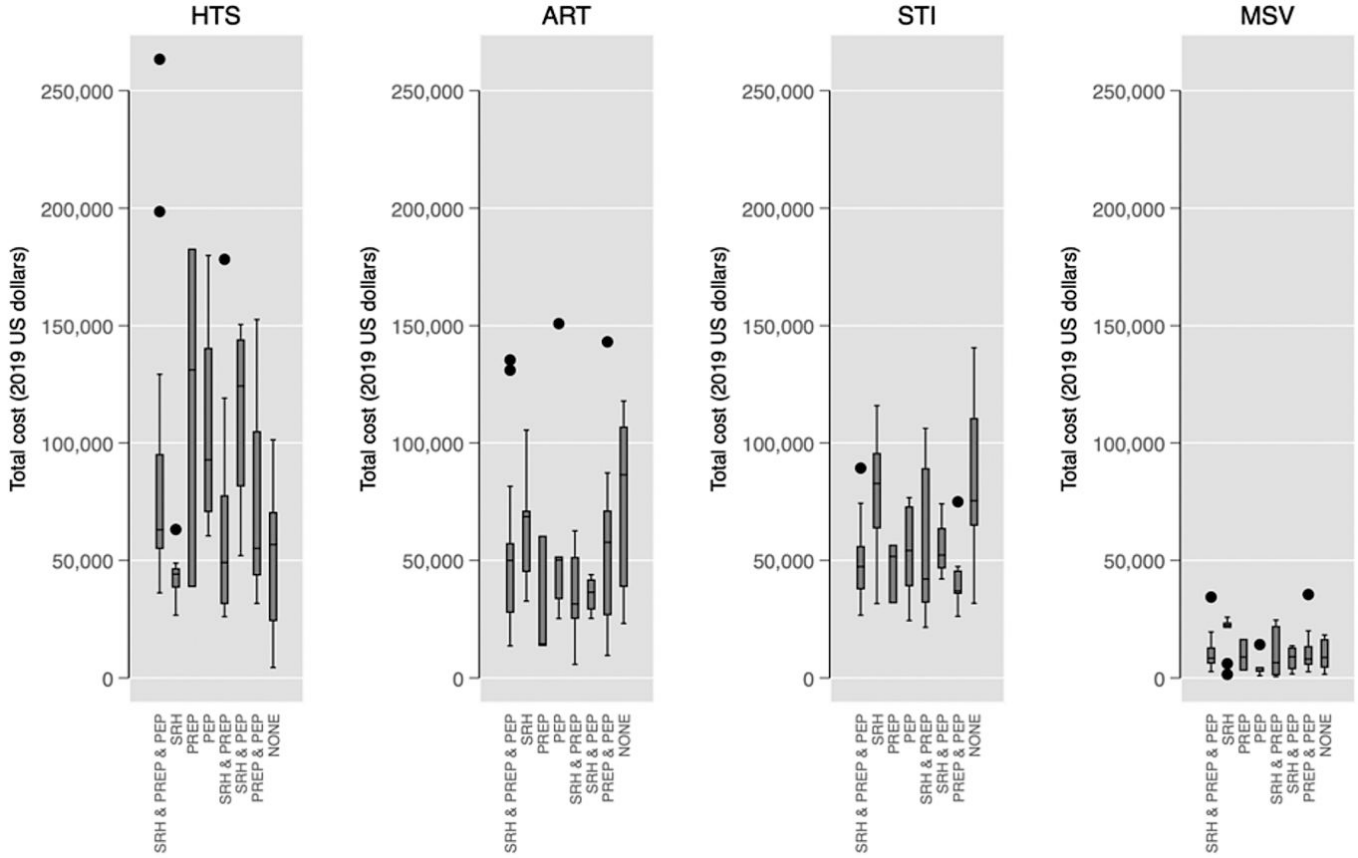
Total costs of HTS, ART, STI screening, and MSV according to whether DICs provided SRH, PrEP, and/or PEP services. Lines inside the boxes indicate the medians; boxes depict inter-quartile range (IQR); whiskers extend to 1.5 times the IQR. *P*-values for the test on the equality of medians across groups are 0.047, 0.106, 0.000, and 0.467 for HTS, ART, STI and MSV total costs, respectively.

Figure 1 displays the distribution of total cost for HTS, ART, STI screening, and MSV, according to DIC provision of SRH, PrEP, and/or PEP services. There were notable and statistically significant differences in median total costs for HTS and STI screening across different service provision subgroups, suggesting that integration of SRH, PrEP, and/or PEP services with HTS and STI screening was related to their total costs.

Table 2 presents the results of our econometric analysis. For HTS, ART, and MSV, we found that our coefficient of interest, $\beta_{1},$ was less than 1, consistent with service-volume gains. Additionally, Supplementary Figure S4 displays the negative relationships between average costs and service scale for each service. Some of the results in Table 2 confirm that service integration was associated with cost-reductions. Cost reductions were associated with service integration in the following cases: HTS costs when provided with STI screening; ART costs when provided with PrEP and PEP services; STI screening costs when combined with ART, PrEP, and PEP; and MSV costs when delivered with ART, SRH, and PrEP services. Service integration coefficients were jointly different from zero in these cases.

**Table 2.** Results from fixed effects log-log regression models of total annual costs.

	Ln HTS total costs	Ln ART total costs	Ln STI total costs	Ln MSV total costs
Ln volume of HTS services	0.657**	−0.072	−0.065	0.208
	(0.279)	(0.081)	(0.115)	(0.256)
Ln volume of ART services	−0.161	0.429***	−0.150***	−0.284*
	(0.096)	(0.069)	(0.050)	(0.163)
Ln volume of STI services	−0.416**	−0.088	0.055	0.211
	(0.190)	(0.079)	(0.114)	(0.265)
Ln volume of MSV services	−0.005	0.027	0.111**	0.274**
	(0.068)	(0.035)	(0.048)	(0.130)
If SRH provided	0.345	0.153	0.285	−0.317
	(0.292)	(0.185)	(0.175)	(0.354)
If PREP provided	−0.301	−0.260	−0.040	0.179
	(0.317)	(0.212)	(0.174)	(0.480)
If PEP provided	0.244	0.099	0.330**	0.630
	(0.272)	(0.153)	(0.150)	(0.519)
If SRH & PREP provided	0.291**	0.135	0.038	−0.797***
	(0.140)	(0.192)	(0.164)	(0.236)
If SRH & PEP provided	−0.046	−0.157	0.093	0.641
	(0.247)	(0.169)	(0.123)	(0.399)
If PREP & PEP provided	−0.144	−0.429***	−0.280***	0.422
	(0.219)	(0.129)	(0.098)	(0.359)
If None (SRH, PREP, PEP) provided	−0.291	0.068	0.097	−0.042
	(0.265)	(0.184)	(0.186)	(0.327)
Intercept	10.995***	9.950***	11.156***	5.464***
	(0.814)	(0.515)	(0.485)	(1.275)
*n*	90	90	90	90
Fixed effects	YES	YES	YES	YES
Price index	YES	YES	YES	YES
*P*-val for joint test^a^ on service integration variables	0.192	0.115	0.001	0.040

Clustered standard errors in parentheses * *P* < 0.1, ** *P* < 0.05, *** *P* < 0.01. Ln means natural logarithm. HTS, HIV testing services; ART, antiretroviral treatment; STI, sexually transmitted infections. SRH, sexual and reproductive health, PrEP, pre-exposure prophylaxis, and PEP, post-exposure prophylaxis. Baseline scenario where all, SRH, PREP, and PEP, services are provided. ^a^The null hypothesis is that coefficients related to scope variables are jointly equal to zero. *n* = number of observations.

Table 3 depicts predicted total costs of HTS, ART, STI screening, and MSV under four potential SRH, PrEP, or PEP service delivery scenarios. These cost predictions, based on the results displayed in Table 2, were calculated by setting all model variables to their mean values (e.g. average total cost of HTS assuming average volumes of ART, STI screening, and MSV services provided). Results indicate that the lowest predicted average total cost of ART, when all factors were constant, occurred when ART was provided along with PrEP and PEP. This also applies to STI screening. For MSV, the lowest total cost occurred when SRH services and PrEP were also provided. For HTS, results show that the lowest average total cost of providing HTS was statistically equal whether PrEP or none of the three services were provided. This conclusion is supported by the equality of means test results shown in Supplementary Table S5, where the *P*-value is 0.9742, suggesting no difference between the two scenarios (PrEP versus None).

**Table 3.** Predicted annual average implementation costs of HTS, ART, STI screening, and MSV under different SRH, PrEP, and PEP service delivery scenarios (2019-USD).

Hypothetical service delivery scenarios	Total HTS cost	Total ART cost	Total STI cost	Total MSV cost
SRH and PREP and PEP	71 166***	50 677***	55 737***	14 623***
	(7.70)	(9.50)	(10.61)	(4.15)
SRH	100 507***	59 066***	74 137***	10 646***
	(4.98)	(11.83)	(10.57)	(4.80)
PREP	52 680***	39 075***	53 572***	17 485*
	(4.46)	(8.24)	(8.32)	(2.27)
PEP	90 828***	55 931***	77 536***	27 462*
	(4.79)	(10.58)	(8.67)	(2.04)
SRH and PREP	95 162***	57 982***	57 890***	6589***
	(9.34)	(11.10)	(11.70)	(4.87)
SRH and PEP	67 992***	43 308***	61 198***	27 756**
	(4.50)	(7.60)	(10.77)	(2.63)
PREP and PEP	61 614***	33 014***	42 124***	22 307**
	(5.82)	(10.90)	(10.55)	(2.60)
NONE	53 199***	54 261***	61 396***	14 016***
	(6.67)	(13.60)	(10.78)	(4.90)
*n*	90	90	90	90

*t* statistics in parentheses *P < 0.05, **P < 0.01, ***P < 0.001. Number of KP tested for HIV, treated for HIV, and screened for STI at their average values. HTS, HIV testing services; ART, antiretroviral treatment; STI, sexually transmitted infections. SRH, sexual and reproductive health, PrEP, pre-exposure prophylaxis, and PEP, post-exposure prophylaxis. *n* = number of observations.

**Figure 2.**
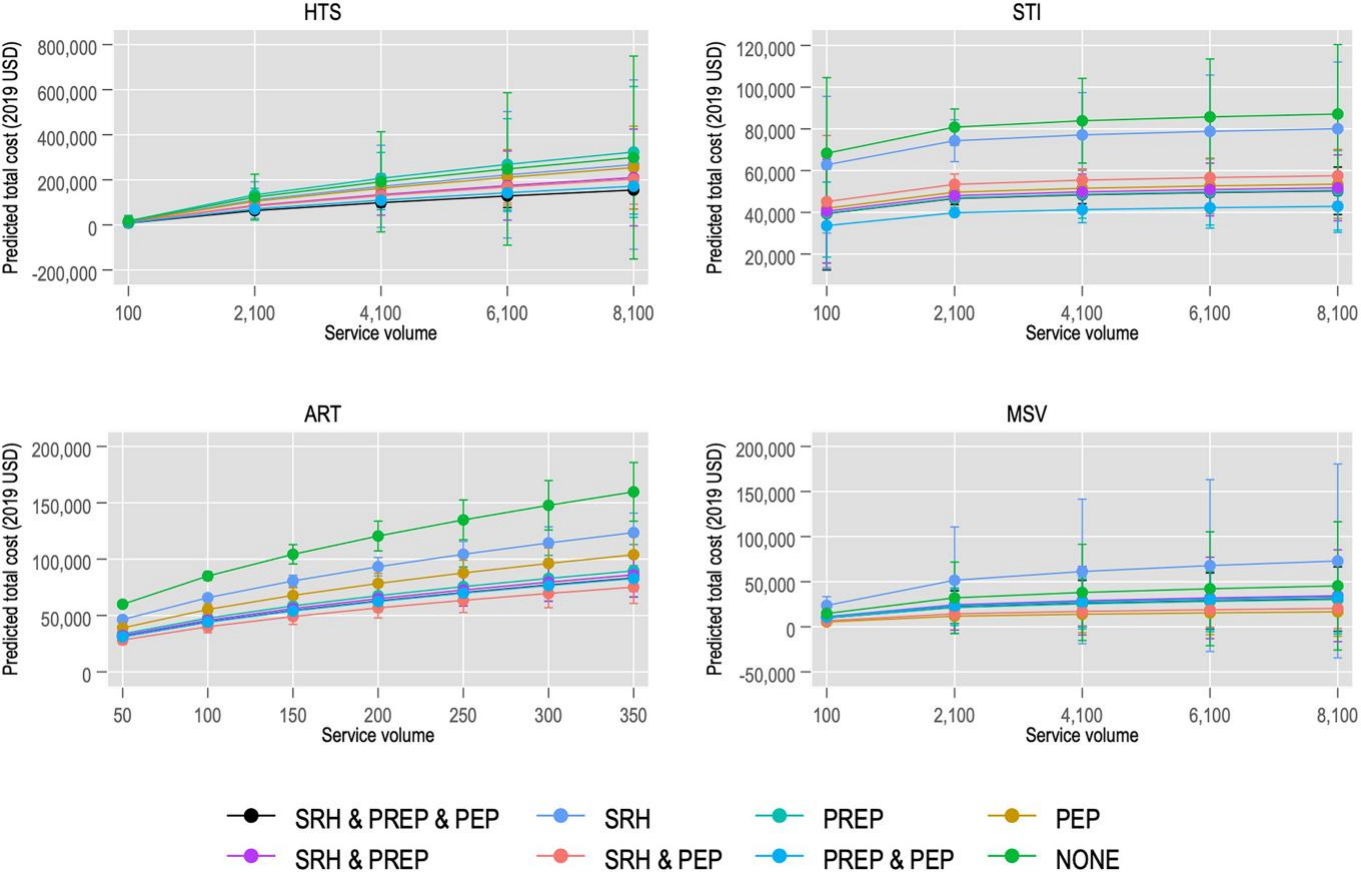
Predicted total annual cost of HTS, ART, STI, and MSV according to whether DICs provide SRH, PrEP, and/or PEP services. 95% confidence intervals shown. Service volume means the number of KP treated for HIV, tested for HIV, screened for STI and intervened for management of sexual violence. All other values are kept at their means when estimating the predicted total costs.

Figure 2 illustrates predicted total costs across different service volumes of HTS, ART, STI screening, and MSV, ranging from minimum to maximum values. The figure shows that throughout the service volume distribution, ART total costs were lowest when SRH and PEP were integrated, while for STI screening, lower total costs were observed when PEP and PrEP were integrated. However, for HTS and MSV, service integration impact across service volume was unclear.

## Discussion

This study explored the relationships between total economic costs, service volume, and integration using data from the LINKAGES program in Kenya and Malawi—a program that provided comprehensive HIV services to KPs. We examined relationships between HTS, ART, STI screening, and MSV total costs and the volume of services delivered across service delivery sites—DICs. We also assessed the total costs of these four services under different SRH, PrEP, and PEP service delivery scenarios. We found two main results. First, service volume increases are correlated with higher total costs, albeit less than proportionally, consistent with possible economies of scale. Second, service integration showed mixed associations with costs: certain combinations of services correlated with cost reductions, but other configurations were associated with cost increases.

Our results on the relationship between service volume and HTS and ART total costs align with earlier studies exploring the association between volume and costs for HIV prevention among KPs (Dandona *et al.* 2005, 2008, Guinness *et al.* 2005, Marseille *et al.* 2007, Chandrashekar *et al.* 2010, Lépine *et al.* 2015, 2016) and ART delivery (Marseille *et al.* 2012, Menzies *et al.* 2012). Theoretically, expanding service volume allows for more efficient distribution of fixed-costs, better use of staff capacity, and enhanced learning, specialization, and innovation (Freeman *et al.* 2021). Our findings suggest that one way to improve the efficiency of KP HIV services could be to expand service coverage, assuming other conditions remain constant. However, we note that the primary objective of DICs is to provide HIV services to KPs. Therefore, potential efficiency improvements through volume increases might counterbalance the distinct advantages of smaller service delivery sites that cater to these marginalized groups. Furthermore, the incremental outreach costs associated with reaching difficult-to-access groups could offset the efficiency benefits of expanding service volume. Therefore, additional avenues to improving efficiency might be relevant.

Our mixed findings on the association between service integration configurations and costs are difficult to compare with previous work, given the absence of similar studies focused on KP HIV services. Previous research on the integration of PrEP services with other general population health services demonstrated cost-effectiveness and affordability (Irungu *et al.* 2019, Roberts *et al.* 2019). While our results indicate potential cost reductions for some KP HIV service combinations, the inconsistent patterns across different integration configurations suggest that economies of scope may depend on the service delivery structures of combined services. For example, cost reductions of ART correlated with integration with PrEP and PEP—services that share pharmaceutical supplies and specialized HIV clinical expertise. These findings suggest the need for context-specific integration strategies rather than universal approaches.

These findings on service volume increases and selective service integration benefits to improve efficiency align well with the current global efforts by international partners to deliver HIV services to KPs through “trusted access platforms” such as the DICs in the LINKAGES program (The Global HIV Prevention Coalition 2020, DiCarlo *et al.* 2022, The Global Fund 2022). These platforms, merging community and clinic service provision, aim to build trust—a cornerstone for engaging KPs. They offer support to address barriers that often prevent KPs from accessing HIV services, including stigma, discrimination, and legal challenges. These platforms serve as the foundation for integrated HIV services for KPs. And they aim to “reach key populations in high numbers” (The Global HIV Prevention Coalition 2020).

This study’s primary strength lies in its unique dataset on comprehensive economic costs of HIV services for KPs covering all activities undertaken at all LINKAGES program implementation levels. We also employed rigorous quantitative methods to analyze statistical relationships between costs, scale and service integration. Using these regression models, we estimated predicted total costs of HIV services under different policy scenarios of service integration.

Several limitations should be considered when interpreting these results. Most importantly, our estimates should not be interpreted causally. Confounders—including service quality and facility management practices—simultaneously affect volume, integration, and costs. Long-term reverse causality may also occur, as cost changes could influence service volume and service integration decisions. We did control for time-invariant unobservable factors and aggregate shocks that might confound the cost–volume relationship, and our fixed-effects approach provides a rigorous identification strategy given the available data constraints. Although the modeling cannot establish causality, our results are consistent with economic theory.

The study’s relatively small sample size is another limitation. Although our study is the largest study examining KP HIV program efficiency in sub-Saharan Africa, it only included 90 observations, which limited the robustness of the econometric analysis. The limited sample size may have contributed to the variability in our integration findings, where cost increases were associated with several integration configurations rather than the expected cost reductions. We also lacked service quality measures—a critical component in efficiency analyses—limiting our ability to assess whether cost differences reflected any variation in service quality.

Several future research priorities emerge from this work. Longitudinal studies could better establish causal relationships while controlling for time-invariant confounders. Larger sample sizes would enable more robust analytical approaches to identify integration effects. Qualitative research examining implementation processes would shed light on operational mechanisms underlying successful integration. Additionally, research incorporating service quality measures, organizational capacity indicators, and client satisfaction metrics would provide essential context for interpreting efficiency findings.

## Conclusion

This research investigated possible avenues for enhancing health service delivery efficiency, focusing on HIV services for KPs. Our study suggests potential strategies to increase the efficiency of HIV-service provision for KPs: expanding service volume and strategically integrating services into service platforms. The insights gleaned from this analysis can inform strategic planning in Kenya, Malawi, and other countries with similar contexts. This is especially critical given declining international health funding. Such strategic orientation might not only optimize resource utilization but also play a critical role in accelerating progress toward HIV control.

## Supplementary Material

czaf067_Supplementary_Data

## Data Availability

The data analyzed in this study are available upon request to SBA.

## Reflexivity statement

The study was born from an ongoing multi-country collaboration on enhancing HIV service delivery for key populations. The results aim to provide key information to support local decision-making. This is an important part of knowledge translation and dissemination from the local setting to other contexts. One goal of research efforts is to disseminate this information to a global audience as well.

## References

[czaf067-B1] Beattie TS, Bhattacharjee P, Suresh M et al Personal, interpersonal and structural challenges to accessing HIV testing, treatment and care services among female sex workers, men who have sex with men and transgenders in Karnataka state, south India. J Epidemiol Community Health 2012;66:ii42–8. 10.1136/jech-2011-200475 22495772

[czaf067-B2] Bernet PM, Singh S. Economies of scale in the production of public health services: an analysis of local health districts in Florida. Am J Public Health 2015;105:S260–7. 10.2105/AJPH.2014.302350 25689207 PMC4355699

[czaf067-B3] Bulstra CA, Hontelez JA, Otto M et al Integrating HIV services and other health services: a systematic review and meta-analysis. PLoS Med 2021;18:e1003836. 10.1371/journal.pmed.1003836 34752477 PMC8577772

[czaf067-B4] Cameron AC, Trivedi PK. Microeconometrics Using Stata, Second. College Station, TX: Stata Press, 2010.

[czaf067-B5] Chandrashekar S, Guinness L, Kumaranayake L et al The effects of scale on the costs of targeted HIV prevention interventions among female and male sex workers, men who have sex with men and transgenders in India. Sex Transm Infect 2010;86:i89–94. 10.1136/sti.2009.038547 20167740 PMC3252618

[czaf067-B6] Dandona L, Kumar SP, Ramesh YK et al Changing cost of HIV interventions in the context of scaling-up in India. AIDS 2008;22:S43–9. 10.1097/01.aids.0000327622.24232.aa 18664952 PMC3688470

[czaf067-B7] Dandona L, Sisodia P, Kumar SP et al HIV prevention programmes for female sex workers in Andhra Pradesh, India: outputs, cost and efficiency. BMC Public Health 2005;5:98. 10.1186/1471-2458-5-98 16181491 PMC1249584

[czaf067-B8] DiCarlo MC, Dallabetta GA, Akolo C et al Adequate funding of comprehensive community-based programs for key populations needed now more than ever to reach and sustain HIV targets. J Int AIDS Soc 2022;25:e25967. 10.1002/jia2.25967 35880969 PMC9318644

[czaf067-B9] Eiling E . *Fast-Track or Off Track: How Insufficient Funding for Key Populations Jeopardises Ending AIDS by* 2030. AIDSFONDS. https://frontlineaids.org/wp-content/uploads/2021/01/Fast-Track-or-Off-Track-report-final.pdf (5 February 2024, date last accessed).

[czaf067-B10] Freeman M, Savva N, Scholtes S. Economies of scale and scope in hospitals: an empirical study of volume spillovers. Manage Sci 2021;67:673–97. 10.1287/mnsc.2019.3572

[czaf067-B11] Getzen TE, Kobernick MS. Health Economics and Financing. New York: John Wiley & Sons, 2022.

[czaf067-B12] Guinness L, Kumaranayake L, Hanson K. A cost function for HIV prevention services: is there a ‘u’—shape?. Cost Eff Resour Alloc 2007;5:13. 10.1186/1478-7547-5-13 17983475 PMC2206005

[czaf067-B13] Guinness L, Kumaranayake L, Rajaraman B et al Does scale matter? The costs of HIV-prevention interventions for commercial sex workers in India. Bull World Health Organ 2005;83:747–55. https://pmc.ncbi.nlm.nih.gov/articles/PMC1852061/ (January 2025, date last accessed). 16283051 PMC1852061

[czaf067-B14] Irungu EM, Sharma M, Maronga C et al The incremental cost of delivering PrEP as a bridge to ART for HIV serodiscordant couples in public HIV care clinics in Kenya. AIDS Res Treat 2019;2019:4170615. 10.1155/2019/4170615 31186955 PMC6521338

[czaf067-B15] Kavanagh MM, Agbla SC, Joy M et al Law, criminalisation and HIV in the world: have countries that criminalise achieved more or less successful pandemic response? BMJ Glob Health 2021;6:e006315. 10.1136/bmjgh-2021-006315 PMC8330576 34341021

[czaf067-B16] Laga M, Vuylsteke B. Evaluating AVAHAN’s design, implementation and impact: lessons learned for the HIV Prevention Community. BMC Public Health 2011;11:S16. 10.1186/1471-2458-11-S6-S16 PMC3287554 22376320

[czaf067-B17] Lépine A, Chandrashekar S, Shetty G et al What determines HIV prevention costs at scale? Evidence from the Avahan Programme in India. Health Econ 2016;25:67–82. 10.1002/hec.3296 26763652 PMC5019264

[czaf067-B18] Lépine A, Vassall A, Chandrashekar S et al Estimating unbiased economies of scale of HIV prevention projects: a case study of Avahan. Social Science & Medicine 2015;131:164–72. 10.1016/j.socscimed.2015.03.007 25779621

[czaf067-B19] Marseille E, Dandona L, Marshall N et al HIV prevention costs and program scale: data from the PANCEA project in five low and middle-income countries. BMC Health Serv Res 2007;7:108. 10.1186/1472-6963-7-108 17626616 PMC1936993

[czaf067-B20] Marseille E, Giganti MJ, Mwango A et al Taking ART to scale: determinants of the cost and cost-effectiveness of antiretroviral therapy in 45 clinical sites in Zambia. PLoS One 2012;7:e51993. 10.1371/journal.pone.0051993 23284843 PMC3527397

[czaf067-B21] McRae S, Brunner JO, Bard JF. Analyzing economies of scale and scope in hospitals by use of case mix planning. Health Care Manag Sci 2020;23:80–101. 10.1007/s10729-019-09476-2 30790146

[czaf067-B22] Menzies NA, Berruti AA, Blandford JM. The determinants of HIV treatment costs in resource limited settings. PLoS One 2012;7:e48726. 10.1371/journal.pone.0048726 23144946 PMC3492412

[czaf067-B23] Obure CD, Jacobs R, Guinness L et al Does integration of HIV and sexual and reproductive health services improve technical efficiency in Kenya and Swaziland? An application of a two-stage semi parametric approach incorporating quality measures. Soc Sci Med 2016;151:147–56. 10.1016/j.socscimed.2016.01.013 26803655 PMC4774477

[czaf067-B24] Opuni M, Figueroa JL, Sanchez-Morales JE et al The cost of providing comprehensive HIV services to key populations: an analysis of the LINKAGES program in Kenya and Malawi. Glob Health Sci Pract 2023a;11:e2200538. 10.9745/GHSP-D-22-00538 37348941 PMC10285728

[czaf067-B25] Opuni M, Sanchez-Morales JE, Figueroa JL et al Estimating the cost of HIV services for key populations provided by the LINKAGES program in Kenya and Malawi. BMC Health Serv Res 2023b;23:337. 10.1186/s12913-023-09279-w 37016402 PMC10071702

[czaf067-B26] Roberts DA, Barnabas RV, Abuna F et al The role of costing in the introduction and scale-up of HIV pre-exposure prophylaxis: evidence from integrating PrEP into routine maternal and child health and family planning clinics in western Kenya. J Int AIDS Soc 2019;22:e25296. 10.1002/jia2.25296 31328443 PMC6643078

[czaf067-B27] Schneider JE, Miller TR, Ohsfeldt RL et al The economics of specialty hospitals. Med Care Res Rev 2008;65:531–53. 10.1177/1077558708316687 18519817

[czaf067-B28] Siapka M, Remme M, Obure CD et al Is there scope for cost savings and efficiency gains in HIV services? A systematic review of the evidence from low- and middle-income countries. Bull World Health Organ 2014;92:499–511. 10.2471/BLT.13.127639 25110375 PMC4121865

[czaf067-B29] StataCorp . Stata Software 18. College Station, TX: StataCorp LLC, 2023.

[czaf067-B30] Sweeney S, Obure CD, Maier CB et al Costs and efficiency of integrating HIV/AIDS services with other health services: a systematic review of evidence and experience. Sex Transm Infect 2012;88:85–99. 10.1136/sextrans-2011-050199 22158934

[czaf067-B31] The Global Fund . *Technical Brief. HIV Programming at Scale for and with Key Population*. The Global Fund. https://www.theglobalfund.org/media/4794/core_keypopulations_technicalbrief_en.pdf (20 December 2022, date last accessed).

[czaf067-B32] The Global HIV Prevention Coalition . *Key population trusted access platforms.* 2020. https://www.unaids.org/sites/default/files/media_asset/2023-global-hiv-prevention-coalition-scorecards-key-findings_en.pdf.

[czaf067-B33] UNAIDS . *The path that ends AIDS: UNAIDS Global AIDS Update* 2023. 2023. https://www.unaids.org/en/resources/documents/2023/global-aids-update-2023 (25 January 2024, date last accessed).

[czaf067-B34] Vassall A, Chandrashekar S, Pickles M et al Community mobilisation and empowerment interventions as part of HIV prevention for female sex workers in southern India: a cost-effectiveness analysis. PLoS One 2014;9:e110562. 10.1371/journal.pone.0110562 25333501 PMC4204894

[czaf067-B35] Weaver M, Deolalikar A. Economies of scale and scope in Vietnamese hospitals. Soc Sci Med 2004;59:199–208. 10.1016/j.socscimed.2003.10.014 15087154

[czaf067-B36] World Health Organization . *Consolidated guidelines on HIV prevention, diagnosis, treatment and care for key population*. Genève: World Health Organization. https://apps.who.int/iris/handle/10665/260444 (27 September 2021, last date accessed).

[czaf067-B100] Zweifel P, Breyer F, Kifmann M. Hospital services and efficiency, In: Zweifel P, Breyer F, Kifmann M (eds). *Health Economics* [Internet]. Berlin, Heidelberg: Springer, 2009 [cited 2022 Feb 21], 311–29. 10.1007/978-3-540-68540-1_9

