# Supplementary material for: Assessing the cost implications of integrating and scaling up HIV services for key populations in Kenya and Malawi
Source: Health Policy Plan. 2025 Oct 31;41(1):5–12. doi: 10.1093/heapol/czaf067 (PMC12828696; doi:10.1093/heapol/czaf067)
Supplement: czaf067_Supplementary_Data [file czaf067_supplementary_data.docx]

**ADDITIONAL FILES OF**

**“Cost variation in HIV services for key populations in Kenya and Malawi: the role of service scale and scope”**

***Additional file S1: LINKAGES program implementation levels and sample size by level***


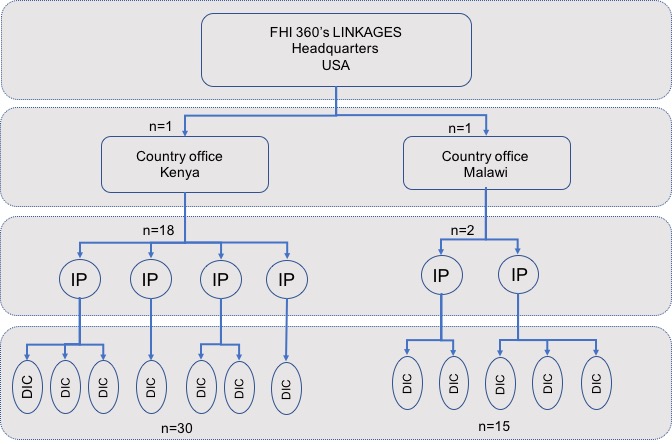


Note: FHI: Family Health International; IP: Implementing partners; DIC: Drop-in centre

***Additional file S2:* Cost categories and sub-categories included in the analysis.**

| **Cost category** | **Sub-categories** | **Source of cost information** |
| --- | --- | --- |
| **Clinical supplies** | - Male condoms, female condoms, and lubricants. - STI treatment drugs: cefixime, doxycycline, acyclovir, metronidazole, clotrimazole, azithromycine, fluconazole, penicillin, ciprofloxacine, erythromycin, gentamycine. - PEP, PrEP, and ART drugs: TDF/3TC/ATVr, ATVr, TDF/3TC, TDF/3TC/EFV, AZT/3TC, etc. - Family planning: intrauterine device, combined oral contraceptive pills, progestogen-only pill, implanon, patch, depo shot, sponge, vaginal ring, cervical cap, emergency pill, diaphragm, spermicide. - HIV tests: HIV rapid test, viral load sample. - Management of sexual violence: emergency pills, PEP drugs, HIV tests. | IP/DIC consumption data report and request |
| **Staff** | Monthly salary, total benefits package, medical insurance and worker compensation, life insurance, social security, pension fund, overtime pay, severance, paid leave, allowances, accommodation, relocation, benefits. | IP/DIC staff payrolls |
| **Peer workers** | Monthly payment for peer educators, peer navigators, and outreach workers. | IP/DIC staff payrolls |
| **Transportation** | Staff per diem, public transportation, rent of vehicles, fuel, vehicle maintenance, vehicle insurance, flights, general transportation, outreach transportation, supervision transportation. | IP/DIC program expense Report/detailed profit and loss account |
| **Other recurrent**  **(Utilities, operations, and external services)** | Electricity consumption, water consumption, diesel for generator, oil/paraffin/kerosene, internet connection, telephone service, rent, building maintenance, building insurance, general office supplies, security services, medical waste management services. | IP/DIC program expense Report/detailed profit and loss account |
| **Equipment** | Type of item bought, computing equipment, cabinets, chairs, desks, office supplies. | IP/DIC asset register |
| **Training** | HIV care & treatment training, outreach workers training, peer educator training, annual review meetings, community advisory meetings, staff training. | IP/DIC program expense Report/detailed profit and loss account |
|  | |  |

Note: STI: Sexually transmitted infections; PEP: Post-Exposure Prophylaxis; PrEP: Pre-Exposure Prophylaxis; ART: Antiretroviral therapy

***Additional file S3: DIC total costs, average costs, and number of services delivered for HTS, ART, and STI services by country.***

|  | **Total cost**  **(2019-USD)** | | | | **Average cost***  **(2019-USD)** | | | | **Number of services provided** | | | |
| --- | --- | --- | --- | --- | --- | --- | --- | --- | --- | --- | --- | --- |
| **Kenya** |  |  |  |  |  |  |  |  |  |  |  |  |
|  | HTS^a^ | ART^b^ | STI^c^ | MSV^d^ | HTS | ART | STI | MSV | HTS | ART | STI | MSV |
| N | 60 | 60 | 60 | 60 | 60 | 60 | 60 | 57 | 60 | 60 | 60 | 60 |
| Mean | 84,707 | 47,548 | 45,758 | 8,644 | 43 | 591 | 23.7 | 240 | 2593 | 109 | 2527 | 107 |
| Median | 65,557 | 42,561 | 42,367 | 7,032 | 31.5 | 455 | 20 | 114 | 2136 | 84 | 2182 | 44 |
| Min | 26,108 | 5,799 | 21,550 | 582 | 10 | 259 | 4.92 | 17.4 | 342 | 2 | 398 | 0 |
| Max | 263,374 | 150,863 | 89,272 | 35,501 | 194 | 3,866 | 79.6 | 1,012 | 9253 | 346 | 6880 | 752 |
| Sd | 49,300 | 30,841 | 14,685 | 6,772 | 33.2 | 499 | 13.9 | 273 | 1745 | 83 | 1467 | 145 |
| **Malawi** |  |  |  |  |  |  |  |  |  |  |  |  |
| N | 30 | 30 | 30 | 30 | 30 | 29 | 30 | 30 | 30 | 29 | 30 | 30 |
| Mean | 47,776 | 71,940 | 87,897 | 15,193 | 119 | 1,378 | 70.1 | 1,620 | 480 | 116 | 1765 | 24 |
| Median | 45,392 | 70,935 | 87,034 | 16,955 | 85.3 | 646 | 54.8 | 896 | 460 | 105 | 1421 | 20 |
| Min | 4,452 | 23,227 | 37,962 | 1,947 | 31.2 | 375 | 25.1 | 111 | 76 | 5 | 538 | 1 |
| Max | 101,340 | 117,960 | 140,580 | 25,846 | 332 | 5,785 | 188 | 18,028 | 1045 | 282 | 4325 | 115 |
| Sd | 22,452 | 29,860 | 23,307 | 7,898 | 82.6 | 1,486 | 48.5 | 3,323 | 218 | 84 | 1098 | 22 |

Notes: N= number of observations. Min=minimum value. Max=maximum value. Sd=Standard deviation. The average cost was calculated by dividing the total cost by the number of services provided. ^a^ Number of HIV tests delivered to KPs. ^b^ Number of KPs who received antiretroviral treatment. ^c^ Number of STI screening services delivered to KPs. ^d^ Number of management of sexual violence services provided to KPs. FSW=Female sex workers. MSM=Men who have sex with men. MSW= Male sex workers.

***Additional file S4: Relationship between HTS, ART, STI and MSV service average costs and service scale***

***
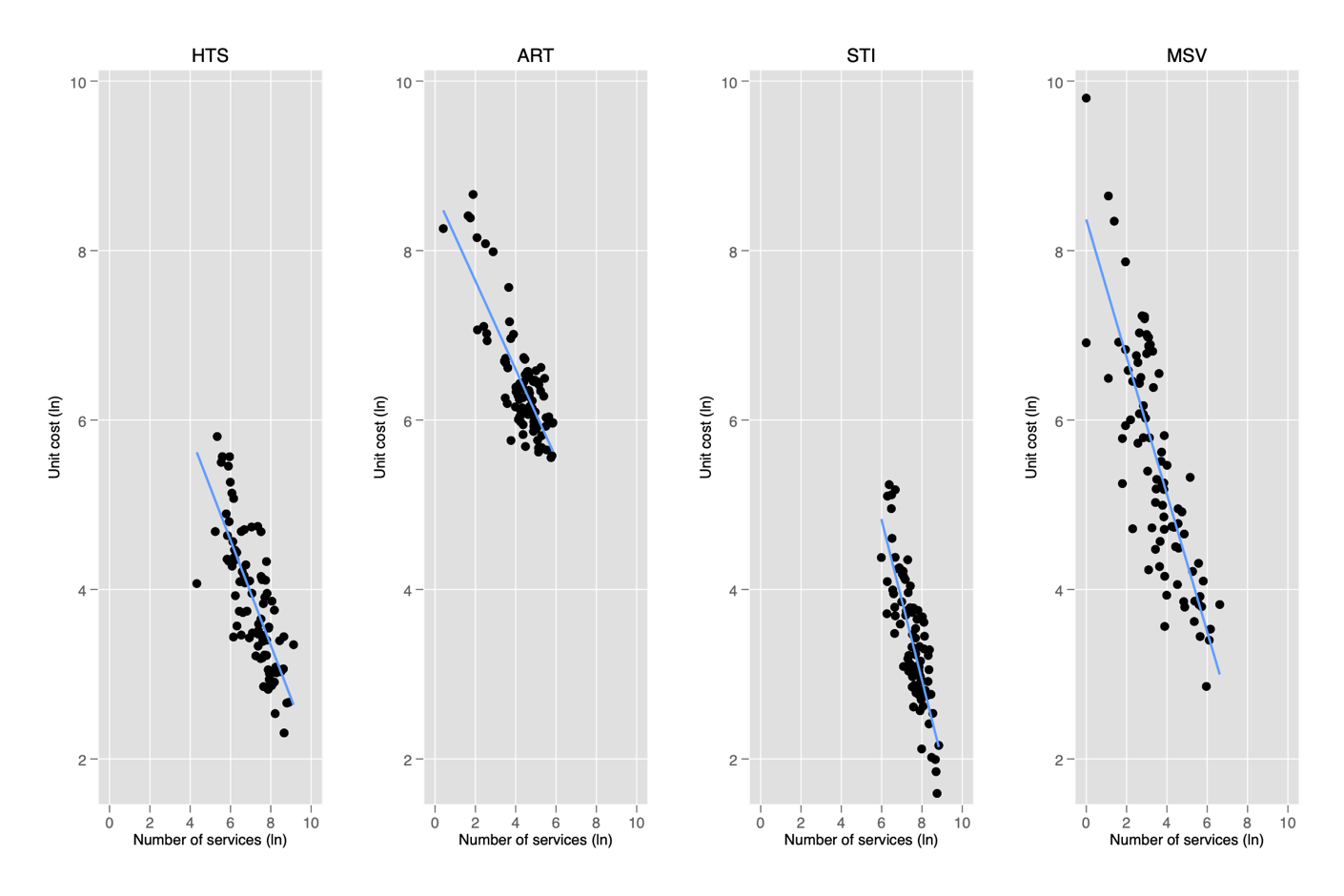
***

Notes: Fit lines are derived by predicting total cost using a regression model using the number of services as the explanatory variable.

***Additional file S5: P-values associated with statistical pair-wise differences across predicted total costs.***

| **Policy scenarios** | **HTS model** | **ART model** | **STI model** | **MSV model** |
| --- | --- | --- | --- | --- |
| SRH&PREP&PEP versus SRH | 0.2330 | 0.3662 | **0.0817** | 0.3517 |
| SRH&PREP&PEP versus PREP | 0.2812 | 0.1836 | 0.8049 | 0.7062 |
| SRH&PREP&PEP versus PEP | 0.3604 | 0.4846 | **0.0249** | 0.3190 |
| SRH&PREP&PEP versus SRH&PREP | **0.0230** | 0.4458 | 0.8027 | **0.0080** |
| SRH&PREP&PEP versus SRH&PEP | 0.8396 | 0.3061 | 0.4104 | 0.1813 |
| SRH&PREP&PEP versus PREP&PEP | 0.4687 | **0.0010** | **0.0030** | 0.2993 |
| SRH&PREP&PEP versus NONE | 0.2324 | 0.6851 | 0.5734 | 0.8889 |
| SRH versus PREP | **0.0782** | **0.0052** | **0.0746** | 0.4213 |
| SRH versus PEP | 0.7540 | 0.7115 | 0.8084 | 0.2166 |
| SRH versus SRH&PREP | 0.8155 | 0.7957 | **0.0063** | **0.0964** |
| SRH versus SRH&PEP | 0.2268 | **0.0537** | 0.2462 | 0.1213 |
| SRH versus PREP&PEP | 0.1437 | **0.0002** | **0.0016** | 0.2097 |
| SRH versus NONE | **0.0245** | 0.2389 | 0.1132 | 0.2715 |
| PREP versus PEP | **0.0653** | **0.0039** | **0.0001** | 0.4398 |
| PREP versus SRH&PREP | **0.0169** | **0.0097** | 0.6587 | 0.1603 |
| PREP versus SRH&PEP | 0.4638 | 0.5744 | 0.1579 | 0.2197 |
| PREP versus PREP&PEP | 0.1971 | 0.1158 | **0.0147** | 0.1818 |
| PREP versus NONE | 0.9742 | **0.0160** | 0.4040 | 0.6519 |
| PEP versus SRH&PREP | 0.8357 | 0.8113 | 0.1125 | 0.1107 |
| PEP versus SRH&PEP | 0.3656 | 0.1024 | **0.0385** | 0.9832 |
| PEP versus PREP&PEP | 0.1659 | **0.0000** | **0.0000** | 0.7069 |
| PEP versus NONE | **0.0755** | 0.8182 | 0.1775 | 0.3031 |
| SRH&PREP versus SRH&PEP | 0.1281 | **0.0923** | 0.7232 | **0.0422** |
| SRH&PREP versus PREP&PEP | **0.0326** | **0.0007** | **0.0493** | **0.0631** |
| SRH&PREP versus NONE | **0.0058** | 0.3264 | 0.5829 | **0.0124** |
| SRH&PEP versus PREP&PEP | 0.7517 | 0.1167 | **0.0001** | 0.5392 |
| SRH&PEP versus PREP&PEP | 0.3986 | 0.1496 | 0.9823 | 0.1845 |
| PREP&PEP versus NONE | 0.5962 | **0.0011** | **0.0270** | 0.3391 |

*Notes: The null hypothesis is that estimates are jointly equal to zero. p-values smaller than 0.10 in bold.*
